# Excess dietary sugar and its impact on periodontal inflammation: a narrative review

**DOI:** 10.1038/s41405-024-00265-w

**Published:** 2024-10-09

**Authors:** Shashikiran Shanmugasundaram, Shaswata Karmakar

**Affiliations:** Department of Periodontology, Manipal College of Dental Sciences, Manipal, Manipal Academy of Higher Education, Manipal, Karnataka India

**Keywords:** Nutrition and diet in dentistry, Periodontitis

## Abstract

**Introduction:**

Sugar is omnipresent in the current food environment and sugar consumption has drastically risen over the past century. Extensive evidence highlights the negative health consequences of consuming excess dietary sugars, leading the World Health Organization (WHO) and the American Heart Association (AHA) to devise guidelines to restrict sugar intake. According to the WHO’s Global Oral Health Status Report of 2022, oral diseases and severe periodontitis are a massive public health problem, and dietary sugars are a modifiable risk factor.

**Methods:**

We conducted a literature review using key databases to summarise the health effects of excessive sugar consumption and their potential role in periodontal inflammation.

**Results and conclusion:**

Available evidence suggests that excess dietary fructose and sucrose can cause low-grade systemic inflammation; and induce dysbiosis in both gut and the oral microbiota. Also, dietary sugar is potentially addictive and hypercaloric and its overconsumption can lead to obesity, metabolic syndrome, and other risk factors for periodontal inflammation. Hence, an unbalanced diet with excess dietary sugars holds the potential to initiate and aggravate periodontal inflammation. In the modern food environment that enables and facilitates a high-sugar diet, adopting a diverse diet and restricting sugar intake according to WHO and AHA guidelines seem beneficial to systemic and periodontal health. Since clinical evidence is limited, future research should study the effectiveness of dietary interventions that control sugar consumption in preventing and managing the global public health problem of periodontal inflammation.

## Introduction

Optimal nutrition through a diverse and well-balanced diet is vital to human health. The chronic non-communicable disease (NCD) burden of modern times is largely linked to suboptimal diet and nutrition [1]. Carbohydrates are one of the major macronutrients integral to an optimal diet and their impact on health and disease has been discussed extensively in the scientific literature [2, 3]. Dietary sugars—a widely consumed and important source of carbohydrates—have recently received attention for their impact on human health and disease.

Sugars are simple carbohydrates and can be monosaccharides, disaccharides, or sugar alcohols [4]. Glucose and fructose are the most common monosaccharides. Both glucose and fructose have the same chemistry—C_6_H_12_O_6_— but differ in structure [5, 6]. These monosaccharides naturally occur in fruits in high quantities. Often, fructose is considered the sweetest of the simple sugars (depending on the form of consumption) with a higher relative sweetness over other mono and disaccharides [4, 7–9]. Sucrose is the most common disaccharide predominant in cane and beet sugar and is broken down to its constituent monosaccharides—glucose and fructose—in the intestinal mucosa [10]. Glucose can be readily used by all cells for energy. Dietary fructose is metabolised in the liver, which is either converted to lactate (for oxidation in tissues) or glucose (for use or storage) [11, 12].

In prehistoric times, natural sweetness was only found in energy and nutrient-rich food sources (fruit, dates, and honey). Hence, the pleasant taste of sweetness means an easily digestible, instant source of energy and an absence of harmful toxins. This led to a ubiquitous preference for sweet taste among humans [13, 14]. Sugar was first extracted from cane juice as granular crystals in India around 500 BC [15]. The Industrial Revolution improved the purity of sugar and the efficiency of sugar production, making it a household commodity. Since then, the worldwide consumption of sugar has skyrocketed and continues in the 21st century [16]. In the modern world, sugar is omnipresent in the form of ultra-processed food and sugar-sweetened beverages [17]. They are widely used to enhance the palatability of processed and packaged food and extend its shelf life. An average American consumes 17 teaspoons (approximately 68 grams) of added sugar daily [18, 19]. India is the highest sugar consumer in the world where an average person consumes approximately 18 kilograms of sugar per year [20, 21]. A 2019 study found that in approximately 60% of the population of the United Kingdom, the total energy from free-sugar intake was 12.4% [22].

Consumption of a diet high in sugars is linked to systemic inflammation, gut dysbiosis, metabolic syndrome, and numerous chronic non-communicable diseases (NCDs) of the modern era [23–27]. The American Heart Association (AHA) recommends restricting the consumption of added sugars (sugars added during processing and preparation of food) to less than 36 grams/day for men and to less than 25 grams/day for women [4, 28]. The 2015 World Health Organization (WHO) guideline recommends limiting the intake of free sugars (i.e. total added sugars plus the inherent natural sugars of the food) throughout life and limiting free sugars to less than 10% of the total energy consumption [4, 29].

Oral diseases (a collective term representing tooth decay, periodontitis, tooth loss, and oral cancer) are the most common diseases that affect humans and a global public health problem affecting around 3.5 billion people across the world [30, 31]. The WHO’s Global Oral Health Status Report of 2022 puts the gravity of the problem into perspective; the global prevalence of oral diseases is greater than the combined global prevalence of the five major NCDs (neurodegenerative diseases, type-2 diabetes mellitus, cardiovascular disease, chronic respiratory diseases, and cancer) by a billion cases [31]. The highest number of cases of oral diseases (excluding cancers) are in the Southeast Asian region of the world which is the highest consumer of sugar globally [31, 32]. High sugar consumption, along with tobacco and alcohol abuse, are key modifiable risk factors for preventing oral diseases [30, 31].

Oral diseases and chronic NCDs share several common risk factors and sugar consumption is of particular concern [30, 31]. It has been proposed to redefine the current non-communicable disease framework by including oral disease as an NCD and sugar as a common risk factor for NCDs [24]; the proposed 6×6 approach consists of six NCDs (oral diseases, mental disorders and conditions, cardiovascular disease, diabetes, cancer, and chronic respiratory diseases) with six common risk factors (sugars, air pollution, tobacco, alcohol, unhealthy diet, and sedentary lifestyle) [24].

**Fig. 1:**
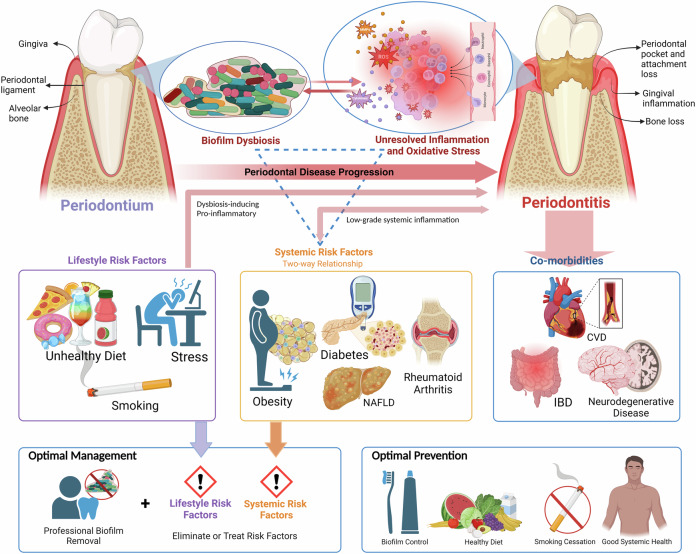
Periodontal inflammation: an overview. The periodontium is the structural apparatus that provides support and anchorage to the tooth. Dysbiosis of the biofilm that accumulates on the tooth elicits an altered immune response in the form of a non-resolving hyperinflammation. Prolonged inflammation and oxidative stress (due to neutrophil oxidative killing) in the periodontium leads to tissue damage and destruction that manifests as periodontitis (pocket formation, attachment loss, and bone loss); gradually the tooth loses support and if inflammation is untreated, results in tooth loss. Periodontitis is influenced by dysbiosis-inducing and pro-inflammatory lifestyle risk factors (unhealthy diet, stress, smoking) and systemic risk factors like obesity, diabetes, rheumatoid arthritis, and non-alcoholic fatty liver disease (NAFLD); periodontitis and systemic risk factors have a two-way relationship through low-grade systemic inflammation. Periodontitis also results in extra-oral co-morbidities such as cardiovascular disease (CVD), inflammatory bowel disease (IBD), and neurodegenerative disease. The optimal management of periodontitis should focus on the removal of dysbiotic biofilm as well as the elimination of dysbiosis-inducing and pro-inflammatory lifestyle and systemic risk factors. Along with daily biofilm control through brushing and flossing, the optimal prevention strategy should also advocate for a healthy diet, smoking cessation, and a healthy lifestyle with good systemic health. (Created in BioRender. Biorender, P. (2024) BioRender.com/x62x671).

Severe periodontitis has a global prevalence of 19%, with a billion cases worldwide in 2019 [31]. There is mounting evidence that a high-sugar, ultra-processed diet increases the risk of periodontal inflammation [33–35]. Periodontitis is the inflammation of tooth-anchoring tissues (the periodontium) due to dysbiosis of the periodontal microbiota, leading to the gradual loss of tooth support (Fig. 1). Periodontal health is inextricably linked to systemic health and several lifestyle factors play an important yet underestimated role in periodontitis [36–38]. Among them, the role of dietary sugars in periodontal inflammation has not been discussed extensively in the literature. Hence, this review aims to summarise the effects of consuming excess dietary sugars (fructose and sucrose) on periodontal inflammation and its relevance in preventing and managing the global public health problem of periodontitis.

## Methods

We adopted a non-systematic, narrative approach to review the existing literature on the health consequences of consuming excess dietary sugars and their potential role in periodontal inflammation. We used the PubMed and SCOPUS databases to search with the keywords “Sugar” OR “Fructose” OR “Sucrose” AND “Inflammation” OR “Periodontal Inflammation” OR “Dysbiosis” OR “Periodontitis” OR “Health”. We summarised the plausible mechanisms relating to excess dietary sugars and systemic and periodontal inflammation using relevant articles.

### Sugar and inflammation

Inflammation is a host defence response against a wide range of noxious stimuli. It is mounted by the immune system, which holds a diverse army of cells and proteins intricately connected in a complex network of physiological and biochemical processes, functioning to protect the host [39, 40]. Unregulated or unresolved chronic inflammation can potentially lead to pathology, making inflammation a double-edged sword [41]. Low-grade systemic chronic inflammation is a common factor in several of the most prevalent NCDs like cardiovascular disease (CVD), diabetes mellitus (DM), neurodegenerative diseases, cancer, and metabolic syndrome [42]. Diet, lifestyle, and microbiome dysbiosis are major contributors to systemic chronic inflammation [42]. Current evidence indicates that excess dietary fructose can induce chronic low-grade systemic inflammation through several mechanisms [23, 43].

#### Inflammation due to gut barrier dysfunction

Permeability is a normal homoeostatic feature of the intestinal mucosal barrier (epithelium and the mucus layer). An increased intestinal permeability disrupts homoeostasis and leads to the transfer of bacteria and their products into the host causing endotoxemia and inflammation [44]. Consuming excess sucrose and fructose increases intestinal permeability and causes endotoxemia and inflammation [45–49]. The consumed fructose in the intestine is absorbed into the hepatic portal vein and reaches the liver for metabolism. Most of the fructose is absorbed into the intestinal epithelial cells through GLUT5 (fructose transporter type 5) and is metabolised by ketohexokinase. This process causes stress in the endoplasmic reticulum and leads to an increased intestinal permeability due to the downregulation of tight junction proteins [43]. Also, a portion of unabsorbed fructose in the small intestine influences the gut microbiota; excess fructose causes an increase in gram-negative bacteria and lipopolysaccharide (LPS) levels in the intestinal environment. LPS activates the macrophages to release pro-inflammatory cytokines that affect the tight junction proteins to cause increased intestinal permeability leading to the entry of endotoxins into the portal vein [45, 50]. The fructose-induced disruption of nitric oxide homoeostasis in the intestinal epithelium also plays a role in the loss of tight junctions and barrier dysfunction [50].

Gut barrier dysfunction causes the bacterial endotoxin to translocate to the liver, activating toll-like receptor 4 (TLR-4) of the Kupffer cells (resident liver macrophages). The activated Kupffer cells, through the nuclear factor kappa-B (NF-κB) pathway, produce pro-inflammatory cytokines like interleukin-1β (IL- 1β) and tumour necrosis factor-α (TNF-α). This cascade causes hepatic insulin resistance and the formation and accumulation of fat in the liver leading to hepatic steatosis and non-alcoholic fatty liver disease (NAFLD) [45, 46, 49–52]. Hepatic steatosis and NAFLD increase circulating pro-inflammatory cytokines like IL-1β, TNF-α, IL-6, and the acute phase C-reactive protein (CRP), leading to chronic low-grade systemic inflammation. The pro-inflammatory state of NAFLD has system-wide effects and is associated with metabolic syndrome, type-2 diabetes mellitus (T2DM), and CVD [53–55]. This pro-inflammatory state triggers the adipose tissue to produce adipokines that further exacerbate NAFLD and systemic inflammation thereby initiating a vicious cycle [53, 54]. Hence, excess dietary sugars can drive inflammation by activating TLR-4 and initiating NAFLD [45, 46, 49–52, 56, 57].

#### Inflammation and oxidative stress due to immune dysfunction

Excessive consumption of fructose is shown to cause inflammation by directly affecting the immune system; a sugar-rich diet increases the production of pro-inflammatory cytokines from macrophages through TLR-4 activation, increases tissue infiltration of macrophages and neutrophils, increases the Th17/Treg cell ratio and promotes inflammation by augmenting Th17 cell-mediated responses [23, 58–60]. Fructose, through AGE-RAGE interactions, causes a pro-inflammatory state [61]; high levels of fructose cause the accumulation of advanced glycation end-products (AGEs) that activate the receptor for advanced glycation end-products (RAGE) and lead to pro-inflammatory dendritic cells that produce IL-1β and IL-6 [62]. Also, high fructose consumption led to systemic oxidative stress in rats; there was an increase in reactive oxygen species (ROS) in blood mononuclear cells and elevated IL-12 and IL-6 in the serum following high fructose consumption for 12 weeks [63].

Metabolites of fructose activate the immune system and elicit pro-inflammatory responses [43, 64]. Uric acid, a byproduct of fructose metabolism, activates nicotinamide adenine dinucleotide oxidase (NADPH oxidase) and increases the production of ROS leading to mitochondrial oxidative stress [43, 65]. This oxidative stress activates the NLRP3 inflammasome which recruits caspase-1 and increases the production of IL-1β [43, 66]. Uric acid also activates the NF-κB pathway to produce pro-inflammatory cytokines [43]. Lactate is another metabolite that accumulates during a high-fructose diet and promotes chronic inflammation [43]. Lactate promotes the formation of the Th17 subset, induces the production of IL-17, and interferes with the killing capacity of CD8^+^ T lymphocytes [43, 67, 68]. High fructose consumption caused the accumulation of NF-κB and IL-1β and reduced the antioxidant capacity in female rats [69]. Overall, high dietary fructose intake induces oxidative stress and low-grade systemic inflammation and puts the body in a pro-inflammatory state [23, 43, 58, 64]. It is important to note that chronic low-grade systemic inflammation and periodontitis have a bi-directional relationship [70, 71].

#### Sugar and periodontal inflammation

A diet high in sugars is associated with periodontal inflammation [33, 34, 72–74]. An observational study found that an excessive intake of added sugars was associated with periodontal disease in adolescents [33]. A recent systematic review concluded that excessive consumption of free sugars was positively associated with periodontal disease, but also reported that the evidence is limited due to the study designs [72]. An analysis of NHANES data (1988–1994) showed that frequent consumption of added sugars was positively associated with periodontal disease [34]. Consuming added sugars beyond the WHO and AHA recommended limit was associated with a higher prevalence of dental decay and inflammatory periodontal disease in Brazilian adolescents [73, 74]. A 2023 systematic review concluded that sugar-sweetened beverage consumption increases the risk of gingival and periodontal inflammation [75]. Another 2023 meta-analysis concluded that restricting free-sugar intake reduces gingival inflammation [76]. Hence, high dietary sugar intake can exacerbate periodontal inflammation and detrimentally affect periodontal health, possibly through low-grade systemic inflammation induced by gut barrier dysfunction, NAFLD and immune dysfunction stemming from a high-sugar diet. Also, gut barrier dysfunction has a bi-directional relationship with periodontal inflammation through the oral-gut axis [77, 78].

### Sugar-induced microbial dysbiosis

The human microbiome is critical to health and disease [79]. “Microbiome” is a term that represents all the microbes that reside in the human body (microbiota) along with their genomic content [80]. Humans have evolved to co-exist with microorganisms symbiotically and live in a finely balanced relationship with the microbiota. The most diverse microbiomes in the human body are in the gut and the oral cavity [81, 82]. A change in the composition and the diversity of the microbiota that leads to the disruption of their symbiotic balance with the host is called dysbiosis [83]. Diet and lifestyle, among several other factors, contribute to the dysbiosis of the microbiota leading to pathological states [84].

#### Gut microbial dysbiosis

The gut microbiota mainly comprises the phyla: *Firmicutes*, *Proteobacteria*, *Bacteroidetes*, *Actinobacteria*, and *Verrucomicrobia* [26, 45]. The gut microbiota produces beneficial short-chain fatty acids (SCFAs) like butyrate and propionate that play an important role in gut barrier integrity and mucosal immunity [85]. Dysbiosis of the gut microbiota has numerous local and systemic consequences and is tied to several systemic diseases [86]. A high-sugar diet induces gut dysbiosis by increasing the abundance of *Bacteroidetes* and *Proteobacteria* and decreasing the abundance of *Firmicutes* and SCFA-producing bacteria [26, 43, 45, 85]. This alteration increases intestinal permeability, endotoxin translocation and inflammation [43, 85, 87]. In rats, 10% fructose feeding for four weeks showed a decrease in *Firmicutes*, an increase in *Bacteroidetes* and *Proteobacteria*, and a decrease in SCFA-producing bacteria [88]. Rats on a sucrose-rich diet showed an increase in the *Bacteroidetes*/*Firmicutes* ratio and a decrease in α diversity. There was also a decrease in SCFAs along with lipid accumulation in the blood and liver [89]. High fructose consumption in rats (10.5g/kg/day) increased the abundance of *Lachnospira*, *Parasutterella*, and *Blantia* with no change in the diversity of the microbiota; increased uric acid levels; increased IL-6 and TNF-α; decreased IL-10; decreased the expression of intestinal tight junction proteins; led to increased intestinal permeability and caused lipid accumulation in the liver [90]. Study participants consuming high-fructose syrup (100g/day) showed increased Bacteroidetes, decreased Firmicutes, and reduced SCFA-producing bacteria [91]. Interestingly, consuming similar levels of fructose (100g/day) entirely through fruits positively affected the gut microbiota [91].

#### Oral microbial dysbiosis

The oral microbiome is a diverse community of around 700 different species of bacteria, predominantly of the phyla: *Actinobacteria*, *Bacteroidetes*, *Firmicutes*, *Fusobacteria*, *Proteobacteria*, *Saccharibacteria*, and *Spirochaetota* [82]. Emerging evidence suggests that excessive intake of dietary sugars can push the oral microbiome towards dysbiosis. Short-term, 10% sucrose rinses (14 days) caused a loss of α-diversity and increased the abundance of *Actinomyces* and *Corynebacterium* in the supragingival microbiota [92]. An in-vitro study showed that 2% sucrose treatment could disrupt the balance between alkali and acid-producing bacteria to cause dysbiosis in a multi-species biofilm model [93]. Also, sucrose incubation reduced oral microbiota diversity and increased the abundance of *Streptococcus* species [94]. Higher sucrose intake and high-glycaemic load food items decreased the oral microbiota diversity in post-menopausal women [95]. A recent systematic review concluded that excessive sugar intake significantly reduced the diversity and caused dysbiosis of the oral microbiota [96]. However, the quality of the systematic review was reportedly questionable [97]. Since the evidence regarding sugars and dysbiosis of the oral microbiota is not robust, future research needs to study further the microbiological consequences of high-sugar intake in the oral cavity.

### The addictive and hypercaloric sugar

#### Addiction potential of sugar

Sweet taste is one of the most intense sensory experiences of humans and current evidence indicates that excess dietary sugars are potentially addictive [98]. Addiction—also known as substance use disorder—is diagnosed using a set of eleven criteria given by the Diagnostic and Statistical Manual of Mental Disorders-5 (DSM-5) [99]. Animal models of sugar addiction display several diagnostic criteria for substance use disorder like bingeing, craving, tolerance, withdrawal, and hazardous use [98, 100, 101]. Also, several parallels exist between sugar and drugs of abuse (cocaine, heroin, amphetamine, nicotine etc); both involve similar cross-sensitization phenomena and neurochemical pathways—Intermittent sugar intake causes the release of dopamine in the nucleus accumbens (the pleasure centre of the brain) that drives a sense of reward (liking; reinforcement and learning; and motivation and wanting) while also delaying the release of satiety-inducing acetylcholine in the nucleus accumbens [98, 100, 101]. Cross-sensitization is when one drug of abuse elicits the same hyperactive locomotory behaviour of another drug of abuse; rats sensitised to amphetamine showed similar hyperactivity when fed with a 10% sucrose solution [102]. Sugar addiction also involves the endogenous opioid system. Highly palatable food rich in sugar releases endogenous opioids in the limbic system and causes dependence; injection of naloxone (an opioid antagonist) causes signs of opioid withdrawal symptoms in sugar-dependent rats [103]. Overall, there is significant evidence to indicate that sugar behaves like a drug and could potentially lead to addiction [98, 100, 101, 104].

#### Overconsumption of sugar

Some evidence indicates that sugars are no different from other calories and that only when sugar consumption results in a positive energy balance (higher energy intake compared to energy expenditure), it leads to obesity, metabolic syndrome and other related co-morbidities [105–111]. However, sugar can be hypercaloric as it is easy to overconsume for several reasons: consuming sugar is potentially addictive; sugar is widely used in most ultra-processed, packaged food that is aggressively and attractively advertised and marketed; sugar and sugar-containing ultra-processed food are hyper-palatable, easily accessible, readily available and relatively inexpensive; sugar and sweets are widely accepted to the extent that it is even associated with celebratory occasions; and unlike nicotine or alcohol, there is neither taboo nor governmental restriction policies on sugar. Another factor that can lead to overconsumption is the lack of adequate satiety after consuming sugar-sweetened beverages [111, 112]. Consuming added sugars can also promote nutrient and energy deficits through various mechanisms leading to overconsumption and obesity [113]. These factors predispose an individual to overconsume added sugars through an unbalanced diet and disrupt the overall energy balance. This paradigm is evident with the global increase in the prevalence of obesity and metabolic syndrome in parallel with the global rise in sugar consumption [114–116]. Extensive evidence in scientific literature shows that sugar is associated with several NCDs [117]. A 2023 umbrella review concluded that high sugar intake is associated with obesity, metabolic syndrome, NAFLD, T2DM, CVD, and cancer [118]. It is important to note that obesity, metabolic syndrome, T2DM, and NAFLD are well-known risk factors with a bi-directional relationship to periodontal inflammation [71, 119–125].

### Clinical relevance

**Fig. 2:**
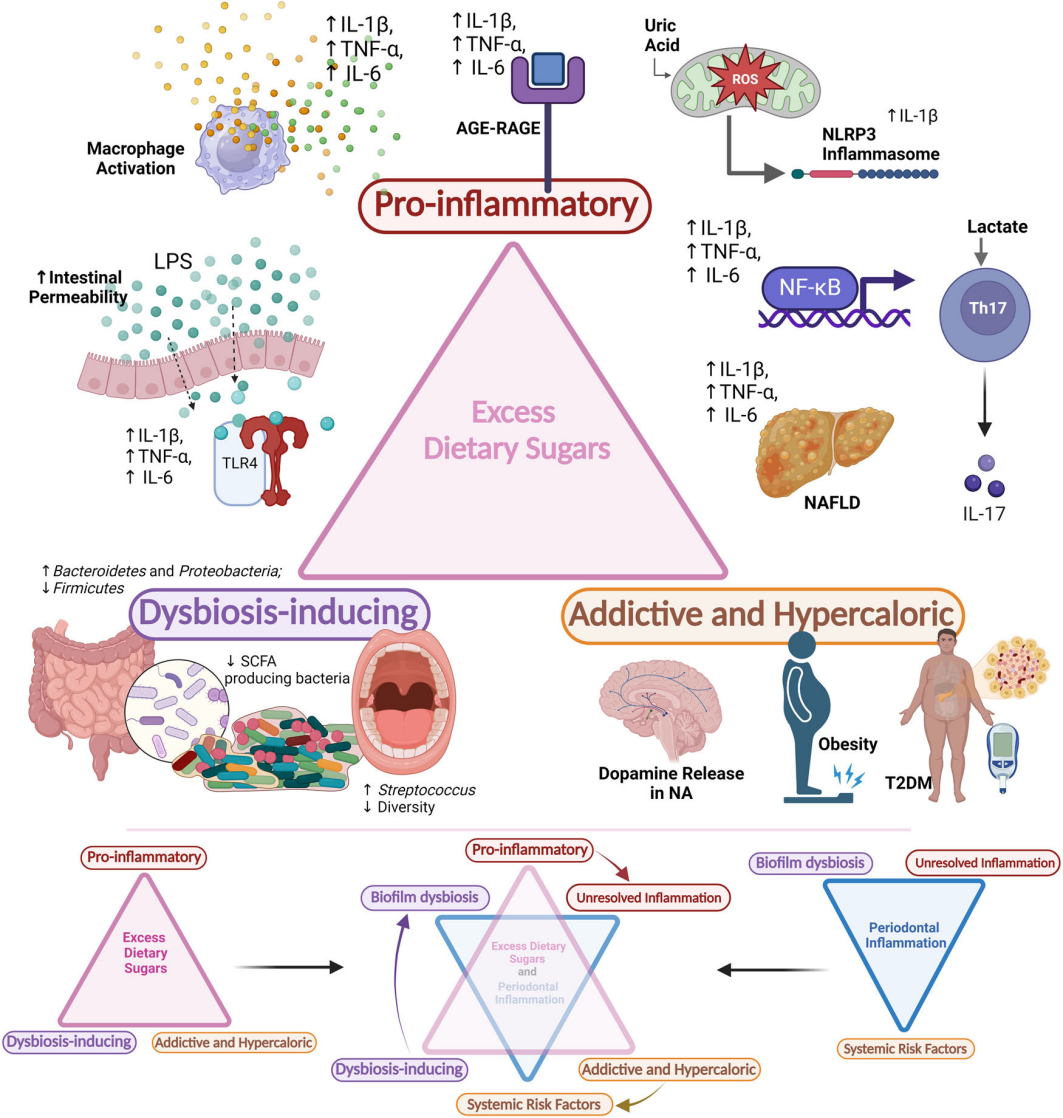
Excess dietary sugars and periodontal inflammation. Excess dietary sugars are potentially pro-inflammatory, dysbiosis-inducing, addictive, and hypercaloric and can initiate and aggravate periodontal inflammation. (AGE- advanced glycation end-products; RAGE- receptor for advanced glycation end-products; ROS- reactive oxygen species; LPS- lipopolysaccharide; TLR-4- toll-like receptor-4; NAFLD- Non-alcoholic fatty liver disease; NF-κB- nuclear factor kappa-B; SCFA- short-chain fatty acids; NA- Nucleus accumbens; T2DM- type-2 diabetes mellitus; IL- interleukin; TNF- α- tumour necrosis factor-α; NLRP3- NOD-, LRR- and pyrin domain-containing protein 3) ( Created in BioRender. Biorender, P. (2024) BioRender.com/h79u248).

There are three critical factors in the initiation, development, and progression of periodontal inflammation: dysbiosis of biofilm communities that initiate periodontal inflammation, unresolved and exaggerated host inflammatory response that causes tissue damage, and systemic diseases and conditions that aggravate the periodontal host inflammatory response [126, 127]. Excessive intake of dietary sugars (fructose and sucrose) can negatively influence all three factors and can play an important role in the initiation, development and progression of periodontal inflammation (Fig. 2)—excess sugars disrupt the eubiotic balance of the oral microbiota to cause dysbiosis; excess dietary fructose is pro-inflammatory and cause low-grade systemic inflammation and oxidative stress that aggravates periodontitis; excess dietary sugars are addictive and hypercaloric which leads to systemic risk factors (obesity, metabolic syndrome, T2DM, and NAFLD) that further aggravate periodontal inflammation.

At present, the main strategies to prevent (daily brushing of teeth) and manage (professional biofilm removal) periodontal inflammation are unidimensional and only aim to control and remove the dysbiotic biofilm on the teeth. Along with the current convention, we need to adopt multidimensional strategies to modify lifestyle factors that induce dysbiosis and a systemic pro-inflammatory state for optimal prevention and management of periodontal inflammation (Fig. 1) [36]. Excess consumption of sugar through an unhealthy diet facilitated by the current food environment seems to be deleterious to systemic and periodontal health and is a modifiable lifestyle risk factor that can be used to prevent and manage periodontal inflammation.

Recently, several dietary and nutritional interventions have been discussed and investigated against periodontal inflammation [128]—a micronutrient-rich diet low in sugar and processed carbohydrates significantly reduced gingival and periodontal inflammation [129, 130]; a similar type of diet also reduced the abundance of periodontitis-associated bacterial species in the supragingival biofilm [131]. A more recent secondary analysis of four studies found that avoiding sugar, processed food, and sweetened beverages for four weeks with a focus on whole food rich in dietary fibre and micronutrients, significantly reduced the inflammation in the gingiva [132].

There is limited research in the literature regarding the effects of sugar and a sugar-rich diet on periodontal health and disease. This lack of a robust body of evidence sets the stage for future sugar and periodontal health research. Also, with the current genomic technologies, future nutrigenomics research could explore the association between sugar and periodontal inflammation in greater detail by studying the effect of excess dietary sugars on gene expression [133].

### Concluding remarks

Available evidence suggests that excessive consumption of sugar is potentially pro-inflammatory and dysbiosis-inducing. Sugar is also potentially addictive, and several factors facilitate its overconsumption leading to systemic risk factors for periodontal inflammation. Current research, although limited, indicates that excessive dietary sugar intake is associated with periodontal inflammation and can be viewed as a modifiable lifestyle risk factor. Lifestyle modification strategies through dietary and nutritional interventions that control sugar and ultra-processed food consumption seem beneficial to periodontal health and need further investigation. Advocating for conscious eating with moderation in dietary sugar intake could serve as a simple, cost-effective, and practical public health strategy for preventing and managing periodontal inflammation.

## References

[1] Gropper SS. The role of nutrition in chronic disease. Nutrients. 2023; 15. 10.3390/NU15030664. 10.3390/nu15030664 PMC9921002 36771368

[2] Clemente-Suárez VJ, Mielgo-Ayuso J, Martín-Rodríguez A, Ramos-Campo DJ, Redondo-Flórez L, Tornero-Aguilera JF. The burden of carbohydrates in health and disease. Nutrients. 2022; 14. 10.3390/NU14183809. 10.3390/nu14183809 PMC9505863 36145184

[3] Ludwig DS, Hu FB, Tappy L, Brand-Miller J. Dietary carbohydrates: role of quality and quantity in chronic disease. BMJ. 2018; 361: 2340. 10.1136/bmj.k2340 PMC5996878 29898880

[4] Cummings JH, Stephen AM. Carbohydrate terminology and classification. Eur J Clin Nutr. 2007;61:S5–S18. 17992187 10.1038/sj.ejcn.1602936

[5] 3d Model of Beta Fructose - American Chemical Society. https://www.acs.org/education/resources/undergraduate/chemistryincontext/interactives/nutrition/3d-model-beta-fructose.html (accessed 19 Mar2024).

[6] 3d Model of Alpha Glucose - American Chemical Society. https://www.acs.org/education/resources/undergraduate/chemistryincontext/interactives/nutrition/3d-model-alpha-glucose.html (accessed 19 Mar2024).

[7] Merino B, Fernández-Díaz CM, Cózar-Castellano I, Perdomo G. Intestinal fructose and glucose metabolism in health and disease. Nutrients. 2020; 12. 10.3390/NU12010094. 10.3390/nu12010094 PMC7019254 31905727

[8] Hanover LM, White JS. Manufacturing, composition, and applications of fructose. Am J Clin Nutr. 1993;58:724S–732S. 8213603 10.1093/ajcn/58.5.724S

[9] Fontvieille AM, Faurion A, Helal I, Rizkalla SW, Falgon S, Letanoux M, et al. Relative sweetness of fructose compared with sucrose in healthy and diabetic subjects. Diabetes Care. 1989;12:481–6. 2758952 10.2337/diacare.12.7.481

[10] Gray GM, Ingelfinger FJ. Intestinal absorption of sucrose in man: interrelation of hydrolysis and monosaccharide product absorption. J Clin Investig. 1966;45:388–98. 5904556 10.1172/JCI105354 PMC292710

[11] Hannou SA, Haslam DE, McKeown NM, Herman MA. Fructose metabolism and metabolic disease. J Clin Invest. 2018;128:545–55. 29388924 10.1172/JCI96702 PMC5785258

[12] Laughlin MR. Normal roles for dietary fructose in carbohydrate metabolism. Nutrients. 2014;6:3117–29. 25100436 10.3390/nu6083117 PMC4145298

[13] Beauchamp GK. Why do we like sweet taste: a bitter tale? Physiol Behav. 2016;164:432–7. 27174610 10.1016/j.physbeh.2016.05.007 PMC5003684

[14] Reed DR, McDaniel AH. The human sweet tooth. BMC Oral Health. 2006;6:1–13. 16934118 10.1186/1472-6831-6-S1-S17 PMC2147592

[15] Gopal L. Sugar-making in ancient India. J Economic Soc Hist Orient. 1964;7:57–72.

[16] Cordain L, Eaton SB, Sebastian A, Mann N, Lindeberg S, Watkins BA, et al. Origins and evolution of the Western diet: health implications for the 21st century. Am J Clin Nutr. 2005;81:341–54. 15699220 10.1093/ajcn.81.2.341

[17] White JR. Sugar. Clin Diabetes. 2018;36:74–76. 29382983 10.2337/cd17-0084 PMC5775006

[18] Lee SH, Park S, Blanck HM. Consumption of added sugars by states and factors associated with added sugars intake among US adults in 50 states and the district of Columbia—2010 and 2015. Nutrients. 2023; 15. 10.3390/NU15020357. 10.3390/nu15020357 PMC9863459 36678228

[19] Lee SH, Zhao L, Park S, Moore LV, Hamner HC, Galuska DA, et al. High added sugars intake among US adults: characteristics, eating occasions, and top sources, 2015–2018. Nutrients. 2023; 15. 10.3390/NU15020265. 10.3390/nu15020265 PMC9867287 36678136

[20] Major sugar consumers worldwide by country 2022/2023 | Statista. https://www.statista.com/statistics/496002/sugar-consumption-worldwide/ (accessed 19 Mar2024).

[21] Dasgupta R, Pillai R, Kumar R, Arora NK. Sugar, salt, fat, and chronic disease epidemic in India: is there need for policy interventions? Indian J Community Med. 2015;40:71–4. 25861165 10.4103/0970-0218.153858 PMC4389505

[22] Rauber F, Louzada M, Martinez Steele E, Rezende L, Millett C, Monteiro CA, et al. Ultra-processed foods and excessive free sugar intake in the UK: a nationally representative cross-sectional study. BMJ Open. 2019;9:027546 10.1136/bmjopen-2018-027546. 10.1136/bmjopen-2018-027546 PMC6830631 31662351

[23] Ma X, Nan F, Liang H, Shu P, Fan X, Song X, et al. Excessive intake of sugar: an accomplice of inflammation. Front Immunol. 2022;13:988481 10.3389/FIMMU.2022.988481. 36119103 10.3389/fimmu.2022.988481 PMC9471313

[24] Benzian H, Daar A, Naidoo S. Redefining the non-communicable disease framework to a 6 × 6 approach: incorporating oral diseases and sugars. Lancet Public Health. 2023;8:e899–e904. 37741288 10.1016/S2468-2667(23)00205-0

[25] Mojto V, Singh RB, Gvozdjakova A, Mojtová M, Kucharská J, Jaglan P, et al. Dietary sugar intake and risk of noncommunicable diseases. *The Role of Functional Food Security in Global Health* 2019;287–99.

[26] Garcia K, Ferreira G, Reis F, Viana S. Impact of dietary sugars on gut microbiota and metabolic health. Diabetology. 2022;3:549–60.

[27] Epner M, Yang P, Wagner RW, Cohen L. Understanding the link between sugar and cancer: an examination of the preclinical and clinical evidence. Cancers (Basel). 2022; 14. 10.3390/CANCERS14246042. 10.3390/cancers14246042 PMC9775518 36551528

[28] Added Sugars | American Heart Association. https://www.heart.org/en/healthy-living/healthy-eating/eat-smart/sugar/added-sugars (accessed 19 Mar2024).

[29] WHO. Guideline. Sugar intake for adults and children. Available online: https://apps.who.int/iris/bitstream/handle/10665/149782/9789241549028_eng.pdf;jsessionid=EFC3B0F9BB09F52DD42BF59E35690A81?sequence=1.

[30] Peres MA, Macpherson L, Weyant RJ, Daly B, Venturelli R, Mathur MR, et al. Oral diseases: a global public health challenge. Lancet. 2019;394:249–60. 31327369 10.1016/S0140-6736(19)31146-8

[31] The Global Status Report on Oral Health 2022. https://www.who.int/team/noncommunicable-diseases/global-status-report-on-oral-health-2022 (accessed 21 Apr2024).

[32] Solomon S, Li YR. Editorial-the sugar industry of Asian region. Sugar Tech. 2016;18:557–8.

[33] Moreira ARO, Batista R, Ladeira L, Thomaz E, Alves C, Saraiva MC, et al. Higher sugar intake is associated with periodontal disease in adolescents. Clin Oral Investig. 2021;25:983–91. 32519237 10.1007/s00784-020-03387-1

[34] Lula ECO, Ribeiro CCC, Hugo FN, Alves CMC, Silva AAM. Added sugars and periodontal disease in young adults: An analysis of NHANES III data. Am J Clin Nutr. 2014;100:1182–7. 25240081 10.3945/ajcn.114.089656

[35] Cassiano LS, Peres MA, Motta J, Demarco FF, Horta BL, Ribeiro CC, et al. Periodontitis is associated with consumption of processed and ultra-processed foods: findings from a population-based study. Nutrients. 2022; 14. 10.3390/NU14183735. 10.3390/nu14183735 PMC9503140 36145111

[36] Shanmugasundaram S, Nayak N, Karmakar S, Chopra A, Arangaraju R. Evolutionary history of periodontitis and the oral microbiota—lessons for the future. Curr Oral Health Rep. 2024;11:1–12.

[37] Chen X, Sun J, Zeng C, Jin F, Ma S, Song J, et al. Association between life’s essential 8 and periodontitis: a population-based study. BMC Oral Health. 2024;24:19 10.1186/S12903-023-03816-Z. 38178120 10.1186/s12903-023-03816-z PMC10768279

[38] Woelber JP, Al-Ahmad A, Alt KW. On the pathogenicity of the oral biofilm: a critical review from a biological, evolutionary, and nutritional point of view. Nutrients. 2022; 14. 10.3390/NU14102174. 10.3390/nu14102174 PMC9144701 35631315

[39] Bennett JM, Reeves G, Billman GE, Sturmberg JP. Inflammation-nature’s way to efficiently respond to all types of challenges: Implications for understanding and managing ‘the epidemic’ of chronic diseases. Front Med (Lausanne). 2018;5:410822. 10.3389/fmed.2018.00316 PMC6277637 30538987

[40] Medzhitov R. Inflammation 2010: new adventures of an old flame. Cell. 2010;140:771–6. 20303867 10.1016/j.cell.2010.03.006

[41] Gupta SC, Kunnumakkara AB, Aggarwal S, Aggarwal BB. Inflammation, a double-edge sword for cancer and other age-related diseases. Front Immunol. 2018;9:2160. 30319623 10.3389/fimmu.2018.02160 PMC6170639

[42] Furman D, Campisi J, Verdin E, Carrera-Bastos P, Targ S, Franceschi C, et al. Chronic inflammation in the etiology of disease across the life span. Nat Med. 2019;25:1822–32. 31806905 10.1038/s41591-019-0675-0 PMC7147972

[43] Cheng H, Zhou J, Sun Y, Zhan Q, Zhang D. High fructose diet: a risk factor for immune system dysregulation. Hum Immunol. 2022;83:538–46. 35414462 10.1016/j.humimm.2022.03.007

[44] Bischoff SC, Barbara G, Buurman W, Ockhuizen T, Schulzke JD, Serino M, et al. Intestinal permeability - a new target for disease prevention and therapy. BMC Gastroenterol. 2014;14:1–25. 25407511 10.1186/s12876-014-0189-7 PMC4253991

[45] Guney C, Bal NB, Akar F. The impact of dietary fructose on gut permeability, microbiota, abdominal adiposity, insulin signaling and reproductive function. Heliyon. 2023;9:18896 10.1016/J.HELIYON.2023.E18896. 10.1016/j.heliyon.2023.e18896 PMC10447940 37636431

[46] Arnone D, Chabot C, Heba AC, Kökten T, Caron B, Hansmannel F, et al. Sugars and gastrointestinal health. Clin Gastroenterol Hepatol. 2022;20:1912–1924.e7. 34902573 10.1016/j.cgh.2021.12.011

[47] Cho YE, Kim DK, Seo W, Gao B, Yoo SH, Song BJ. Fructose promotes leaky gut, endotoxemia, and liver fibrosis through ethanol-inducible cytochrome P450-2E1–mediated oxidative and nitrative stress. Hepatology. 2021;73:2180–95. 30959577 10.1002/hep.30652 PMC6783321

[48] Guo P, Wang H, Ji L, Song P, Ma X. Impacts of fructose on intestinal barrier function, inflammation and microbiota in a piglet model. Nutrients. 2021;13. 10.3390/NU13103515. 10.3390/nu13103515 PMC8541567 34684516

[49] Bischoff SC, Kaden-Volynets V, Filipe Rosa L, Guseva D, Seethaler B. Regulation of the gut barrier by carbohydrates from diet – Underlying mechanisms and possible clinical implications. Int J Med Microbiol. 2021;311:151499. 33864957 10.1016/j.ijmm.2021.151499

[50] Staltner R, Burger K, Baumann A, Bergheim I. Fructose: a modulator of intestinal barrier function and hepatic health? Eur J Nutr. 2023;62:3113–24. 37596353 10.1007/s00394-023-03232-7 PMC10611622

[51] Jensen T, Abdelmalek MF, Sullivan S, Nadeau KJ, Green M, Roncal C, et al. Fructose and sugar: a major mediator of nonalcoholic fatty liver disease. J Hepatol. 2018;68:1063–75. 29408694 10.1016/j.jhep.2018.01.019 PMC5893377

[52] Huneault HE, Tovar AR, Sanchez-Torres C, Welsh JA, Vos MB. The impact and burden of dietary sugars on the liver. Hepatol Commun. 2023; 7. 10.1097/HC9.0000000000000297. 10.1097/HC9.0000000000000297 PMC10629746 37930128

[53] Gehrke N, Schattenberg JM. Metabolic inflammation-a role for hepatic inflammatory pathways as drivers of comorbidities in nonalcoholic fatty liver disease? Gastroenterology. 2020;158:1929–1947.e6. 32068022 10.1053/j.gastro.2020.02.020

[54] Luo Y, Lin H. Inflammation initiates a vicious cycle between obesity and nonalcoholic fatty liver disease. Immun Inflamm Dis. 2021;9:59–73. 33332766 10.1002/iid3.391 PMC7860600

[55] Asrih M, Jornayvaz FR. Inflammation as a potential link between nonalcoholic fatty liver disease and insulin resistance. J Endocrinol. 2013;218:R25–R36. 23833274 10.1530/JOE-13-0201

[56] Fajstova A, Galanova N, Coufal S, Malkova J, Kostovcik M, Cermakova M, et al. Diet rich in simple sugars promotes pro-inflammatory response via gut microbiota alteration and TLR4 signaling. Cells. 2020;9:2701. 33339337 10.3390/cells9122701 PMC7766268

[57] Jessri M, Hennessey D, Bader Eddeen A, Bennett C, Zhang Z, Yang Q, et al. Added sugar intake and its forms and sources in relation to risk of non-alcoholic fatty liver disease: results from the Tianjin Chronic Low-grade Systemic Inflammation and Health cohort study. Br J Nutr. 2022;129:2094–101. 10.1017/S000711452200277X 36156191

[58] Barbosa P, Carvalho E. Are dietary sugars potent adipose tissue and immune cell modulators? Diabetology. 2023;4:30–45.

[59] Zhang D, Jin W, Wu R, Li J, Park SA, Tu E, et al. High glucose intake exacerbates autoimmunity through reactive oxygen species-mediated TGF-β cytokine activation. Immunity. 2019;51:671–81. 31451397 10.1016/j.immuni.2019.08.001 PMC9811990

[60] Kim HJ, Dinh D, Yang J, Herath K, Seo SH, Son YO, et al. High sucrose intake exacerbates airway inflammation through pathogenic Th2 and Th17 response in ovalbumin (OVA)-induced acute allergic asthma in C57BL/6 mice. J Nutr Biochem. 2024;124:109504. 37944673 10.1016/j.jnutbio.2023.109504

[61] Gugliucci A. Formation of fructose-mediated advanced glycation end products and their roles in metabolic and inflammatory diseases. Adv Nutr. 2017;8:54–62. 28096127 10.3945/an.116.013912 PMC5227984

[62] Jaiswal N, Agrawal S, Agrawal A. High fructose-induced metabolic changes enhance inflammation in human dendritic cells. Clin Exp Immunol. 2019;197:237–49. 30919933 10.1111/cei.13299 PMC6642875

[63] Porto ML, Lírio LM, Dias AT, Batista AT, Campagnaro BP, Mill JG, et al. Increased oxidative stress and apoptosis in peripheral blood mononuclear cells of fructose-fed rats. Toxicol Vitr. 2015;29:1977–81. 10.1016/j.tiv.2015.08.006 26279319

[64] Zhang DM, Jiao RQ, Kong LD. High dietary fructose: Direct or indirect dangerous factors disturbing tissue and organ functions. Nutrients. 2017; 9. 10.3390/nu9040335. 10.3390/nu9040335 PMC5409674 28353649

[65] Lanaspa MA, Sanchez-Lozada LG, Choi YJ, Cicerchi C, Kanbay M, Roncal-Jimenez CA, et al. Uric acid induces hepatic steatosis by generation of mitochondrial oxidative stress: potential role in fructose-dependent and -independent fatty liver. J Biol Chem. 2012;287:40732–44. 23035112 10.1074/jbc.M112.399899 PMC3504786

[66] Joosten LAB, Crişan TO, Bjornstad P, Johnson RJ. Asymptomatic Hyperuricemia – A silent activator of the innate immune system. Nat Rev Rheumatol. 2020;16:75–86. 31822862 10.1038/s41584-019-0334-3 PMC7075706

[67] Haas R, Smith J, Rocher-Ros V, Nadkarni S, Montero-Melendez T, D'Acquisto F, et al. Lactate regulates metabolic and pro-inflammatory circuits in control of T cell migration and effector functions. PLoS Biol. 2015;13:1002202 10.1371/JOURNAL.PBIO.1002202. 10.1371/journal.pbio.1002202 PMC4504715 26181372

[68] Pucino V, Bombardieri M, Pitzalis C, Mauro C. Lactate at the crossroads of metabolism, inflammation, and autoimmunity. Eur J Immunol. 2017;47:14–21. 27883186 10.1002/eji.201646477

[69] Kovačević S, Nestorov J, Matić G, Elaković I. Fructose-enriched diet induces inflammation and reduces antioxidative defense in visceral adipose tissue of young female rats. Eur J Nutr. 2017;56:151–60. 26433940 10.1007/s00394-015-1065-0

[70] Cecoro G, Annunziata M, Iuorio MT, Nastri L, Guida L. Periodontitis, low-grade inflammation and systemic health: a scoping review. Medicina (B Aires). 2020; 56. 10.3390/MEDICINA56060272. 10.3390/medicina56060272 PMC7353850 32486269

[71] Hajishengallis G, Chavakis T. Local and systemic mechanisms linking periodontal disease and inflammatory comorbidities. Nat Rev Immunol. 2021;21:426–40. 33510490 10.1038/s41577-020-00488-6 PMC7841384

[72] Kusama T, Nakazawa N, Takeuchi K, Kiuchi S, Osaka K. Free sugar intake and periodontal diseases: a systematic review. Nutrients. 2022; 14. 10.3390/NU14214444/S1. 10.3390/nu14214444 PMC9656760 36364708

[73] Ladeira LLC, Nascimento GG, Leite F, Alves-Costa S, Thomaz E, Alves C, et al. Sugar intake above international recommendations and oral disease burden: A population-based study. Oral Dis. 2022;30:615–23. 10.1111/odi.14464. 36504466 10.1111/odi.14464

[74] Carmo CDS, Ribeiro M, Teixeira J, Alves C, Franco MM, França A, et al. Added sugar consumption and chronic oral disease burden among adolescents in Brazil. J Dent Res. 2018;97:508–14. 29342369 10.1177/0022034517745326

[75] Gupta V, Dawar A, Bhadauria US, Purohit BM, Nilima N. Sugar-sweetened beverages and periodontal disease: a systematic review. Oral Dis. 2023;29:3078–90. 36062371 10.1111/odi.14368

[76] Woelber JP, Gebhardt D, Hujoel PP. Free sugars and gingival inflammation: a systematic review and meta-analysis. J Clin Periodontol. 2023;50:1188–201. 37246336 10.1111/jcpe.13831

[77] Kunath BJ, De Rudder C, Laczny CC, Letellier E, Wilmes P. The oral–gut microbiome axis in health and disease. Nat Rev Microbiol. 2024;2024:1–15. 10.1038/s41579-024-01075-5 39039286

[78] Huang X, Li Y, Zhang J, Feng Q. Linking periodontitis with inflammatory bowel disease through the oral–gut axis: the potential role of porphyromonas gingivalis. Biomedicines. 2024;12:685. 38540299 10.3390/biomedicines12030685 PMC10968003

[79] Malard F, Dore J, Gaugler B, Mohty M. Introduction to host microbiome symbiosis in health and disease. Mucosal Immunol. 2020;14:547–54. 33299088 10.1038/s41385-020-00365-4 PMC7724625

[80] Liu X. Focus: microbiome: microbiome. Yale J Biol Med. 2016;89:275.

[81] Kilian M, Chapple IL, Hannig M, Marsh PD, Meuric V, Pedersen AM, et al. The oral microbiome – an update for oral healthcare professionals. Br Dent J. 2016;221:657–66. 27857087 10.1038/sj.bdj.2016.865

[82] Baker JL, Mark Welch JL, Kauffman KM, McLean JS, He X. The oral microbiome: diversity, biogeography and human health. Nat Rev Microbiol. 2023;22:89–104. 37700024 10.1038/s41579-023-00963-6 PMC11084736

[83] Hou K, Wu ZX, Chen XY, Wang JQ, Zhang D, Xiao C, et al. Microbiota in health and diseases. Signal Transduct Target Ther. 2022;7:1–28. 35461318 10.1038/s41392-022-00974-4 PMC9034083

[84] Martinez JE, Kahana DD, Ghuman S, Wilson HP, Wilson J, Kim S, et al. Unhealthy lifestyle and gut dysbiosis: a better understanding of the effects of poor diet and nicotine on the intestinal microbiome. Front Endocrinol (Lausanne). 2021;12:667066. 34168615 10.3389/fendo.2021.667066 PMC8218903

[85] Jung S, Bae H, Song WS, Jang C. Dietary fructose and fructose-induced pathologies. Annu Rev Nutr. 2022;42:45–66. 35995049 10.1146/annurev-nutr-062220-025831 PMC9904196

[86] Afzaal M, Saeed F, Shah YA, Hussain M, Rabail R, Socol CT, et al. Human gut microbiota in health and disease: Unveiling the relationship. Front Microbiol. 2022;13:999001. 36225386 10.3389/fmicb.2022.999001 PMC9549250

[87] Satokari R. High intake of sugar and the balance between pro- and anti-inflammatory gut bacteria. Nutrients. 2020;12:1348. 32397233 10.3390/nu12051348 PMC7284805

[88] Song M. Dietary fructose induced gut microbiota dysbiosis is an early event in the onset of metabolic phenotype. FASEB J. 2019;33:723.2–723.2.

[89] Sun S, Araki Y, Hanzawa F, Umeki M, Kojima T, Nishimura N, et al. High sucrose diet-induced dysbiosis of gut microbiota promotes fatty liver and hyperlipidemia in rats. J Nutr Biochem. 2021;93:108621 10.1016/J.JNUTBIO.2021.108621. 33705945 10.1016/j.jnutbio.2021.108621

[90] Wang Y, Qi W, Song G, Pang S, Peng Z, Li Y, et al. High-fructose diet increases inflammatory cytokines and alters gut microbiota composition in rats. Mediators Inflamm. 2020;2020:6672636 10.1155/2020/6672636. 33312070 10.1155/2020/6672636 PMC7721508

[91] Beisner J, Gonzalez-Granda A, Basrai M, Damms-Machado A, Bischoff SC. Fructose-induced intestinal microbiota shift following two types of short-term high-fructose dietary phases. Nutrients. 2020;12:1–21. 10.3390/nu12113444 PMC7697676 33182700

[92] Lundtorp Olsen C, Markvart M, Vendius VFD, Damgaard C, Belstrøm D. Short-term sugar stress induces compositional changes and loss of diversity of the supragingival microbiota. J Oral Microbiol. 2023;15:2189770 10.1080/20002297.2023.2189770. 36968295 10.1080/20002297.2023.2189770 PMC10035944

[93] Du Q, Fu M, Zhou Y, Cao Y, Guo T, Zhou Z, et al. Sucrose promotes caries progression by disrupting the microecological balance in oral biofilms: an in vitro study. Sci Rep. 2020;10:1–12. 32076013 10.1038/s41598-020-59733-6 PMC7031525

[94] Onyango SO, De Clercq N, Beerens K, Van Camp J, Desmet T, Van de Wiele T. Oral microbiota display profound differential metabolic kinetics and community shifts upon incubation with sucrose, trehalose, kojibiose, and xylitol. Appl Environ Microbiol. 2020;86:1–14. 10.1128/AEM.01170-20 PMC7414948 32561577

[95] Millen AE, Dahhan R, Freudenheim JL, Hovey KM, Li L, McSkimming DI, et al. Dietary carbohydrate intake is associated with the subgingival plaque oral microbiome abundance and diversity in a cohort of postmenopausal women. Sci Rep. 2022;12:1–12. 35173205 10.1038/s41598-022-06421-2 PMC8850494

[96] Angarita-Díaz M, del P, Fong C, Bedoya-Correa CM, Cabrera-Arango CL. Does high sugar intake really alter the oral microbiota?: A systematic review. Clin Exp Dent Res. 2022;8:1376–90. 35946056 10.1002/cre2.640 PMC9760141

[97] Dhingra K, Jeng JH. Does a high-sugar diet alter the bacterial diversity of the oral cavity? Evid Based Dent. 2023;24:9–11. 10.1038/S41432-023-00862-Y. 36890244 10.1038/s41432-023-00862-y

[98] Avena NM, Rada P, Hoebel BG. Evidence for sugar addiction: behavioral and neurochemical effects of intermittent, excessive sugar intake. Neurosci Biobehav Rev. 2008;32:20–39. 17617461 10.1016/j.neubiorev.2007.04.019 PMC2235907

[99] Hasin DS, O'Brien CP, Auriacombe M, Borges G, Bucholz K, Budney A, et al. DSM-5 criteria for substance use disorders: recommendations and rationale. Am J Psychiatry. 2013;170:834–51. 23903334 10.1176/appi.ajp.2013.12060782 PMC3767415

[100] Wiss DA, Avena N, Rada P. Sugar addiction: From evolution to revolution. Front Psychiatry. 2018;9:385158. 10.3389/fpsyt.2018.00545 PMC6234835 30464748

[101] DiNicolantonio JJ, O’Keefe JH, Wilson WL. Sugar addiction: is it real? A narrative review. Br J Sports Med. 2018;52:910–3. 28835408 10.1136/bjsports-2017-097971

[102] Avena NM, Hoebel BG. Amphetamine-sensitized rats show sugar-induced hyperactivity (cross-sensitization) and sugar hyperphagia. Pharm Biochem Behav. 2003;74:635–9. 10.1016/s0091-3057(02)01050-x 12543229

[103] Colantuoni C, Rada P, McCarthy J, Patten C, Avena NM, Chadeayne A, et al. Evidence that intermittent, excessive sugar intake causes endogenous opioid dependence. Obes Res. 2002;10:478–88. 12055324 10.1038/oby.2002.66

[104] Ahmed SH, Guillem K, Vandaele Y. Sugar addiction: pushing the drug-sugar analogy to the limit. Curr Opin Clin Nutr Metab Care. 2013;16:434–9. 23719144 10.1097/MCO.0b013e328361c8b8

[105] Archer E, Arjmandi B. Falsehoods and facts about dietary sugars: a call for evidence-based policy. Crit Rev Food Sci Nutr. 2021;61:3725–39. 32799555 10.1080/10408398.2020.1804320

[106] Rippe JM, Angelopoulos TJ. Relationship between added sugars consumption and chronic disease risk factors: current understanding. Nutrients. 2016; 8. 10.3390/NU8110697. 10.3390/nu8110697 PMC5133084 27827899

[107] Rippe JM, Angelopoulos TJ. Sugars and health controversies: what does the science say? Adv Nutr. 2015;6:493–503.

[108] Khan TA, Sievenpiper JL. Controversies about sugars: results from systematic reviews and meta-analyses on obesity, cardiometabolic disease and diabetes. Eur J Nutr. 2016;55:25–43. 27900447 10.1007/s00394-016-1345-3 PMC5174149

[109] Morenga LT, Mallard S, Mann J. Dietary sugars and body weight: systematic review and meta-analyses of randomised controlled trials and cohort studies. BMJ. 2012;346:7492 10.1136/BMJ.E7492. 10.1136/bmj.e7492 23321486

[110] Fattore E, Botta F, Agostoni C, Bosetti C. Effects of free sugars on blood pressure and lipids: a systematic review and meta-analysis of nutritional isoenergetic intervention trials. Am J Clin Nutr. 2017;105:42–56. 28003201 10.3945/ajcn.116.139253

[111] Prinz P. The role of dietary sugars in health: molecular composition or just calories? Eur J Clin Nutr. 2019;73:1216–23. 30787473 10.1038/s41430-019-0407-z PMC6760629

[112] Cassady BA, Considine RV, Mattes RD. Beverage consumption, appetite, and energy intake: what did you expect? Am J Clin Nutr. 2012;95:587–93. 22258267 10.3945/ajcn.111.025437 PMC3278240

[113] DiNicolantonio JJ, Berger A. Added sugars drive nutrient and energy deficit in obesity: a new paradigm. Open Heart. 2016;3:e000469. 27547437 10.1136/openhrt-2016-000469 PMC4975866

[114] Malik VS, Hu FB. The role of sugar-sweetened beverages in the global epidemics of obesity and chronic diseases. Nat Rev Endocrinol. 2022;18:205–18. *2022 18:4* 35064240 10.1038/s41574-021-00627-6 PMC8778490

[115] Siervo M, Montagnese C, Mathers JC, Soroka KR, Stephan BCM, Wells JCK. Sugar consumption and global prevalence of obesity and hypertension: an ecological analysis. Public Health Nutr. 2014;17:587–96. 23414749 10.1017/S1368980013000141 PMC10282320

[116] Boutari C, Mantzoros CS. A 2022 update on the epidemiology of obesity and a call to action: as its twin COVID-19 pandemic appears to be receding, the obesity and dysmetabolism pandemic continues to rage on. Metabolism. 2022;133:155217. 35584732 10.1016/j.metabol.2022.155217 PMC9107388

[117] Gillespie KM, Kemps E, White MJ, Bartlett SE. The impact of free sugar on human health—a narrative review. Nutrients. 2023; 15. 10.3390/NU15040889. 10.3390/nu15040889 PMC9966020 36839247

[118] Huang Y, Chen Z, Chen B, Li J, Yuan X, Li J, et al. Dietary sugar consumption and health: umbrella review. BMJ. 2023;381:071609 10.1136/BMJ-2022-071609. 10.1136/bmj-2022-071609 PMC10074550 37019448

[119] Nabila S, Choi J, Kim JE, Hahn S, Hwang IK, Kim TI, et al. Bidirectional associations between periodontal disease and systemic diseases: a nationwide population-based study in Korea. Sci Rep. 2023;13:1–10. 37640779 10.1038/s41598-023-41009-4 PMC10462734

[120] Marruganti C, Suvan JE, D’Aiuto F. Periodontitis and metabolic diseases (diabetes and obesity): tackling multimorbidity. Periodontol. 2023;00:1–16. 2000 10.1111/prd.12536 37845800

[121] Păunică I, Giurgiu M, Dumitriu AS, Păunică S, Pantea Stoian AM, Martu MA, et al. The bidirectional relationship between periodontal disease and diabetes mellitus—a review. Diagnostics. 2023; 13. 10.3390/DIAGNOSTICS13040681. 10.3390/diagnostics13040681 PMC9954907 36832168

[122] Rakita A, Nikolić N, Mildner M, Matiasek J, Elbe-Bürger A. Association between fatty liver index and periodontitis: the korea national health and nutrition examination survey. Sci Rep. 2020;10:1–7. 32123238 10.1038/s41598-020-60797-7 PMC7051950

[123] Sato S, Kamata Y, Kessoku T, Shimizu T, Kobayashi T, Kurihashi T, et al. A cross-sectional study assessing the relationship between non-alcoholic fatty liver disease and periodontal disease. Sci Rep. 2022;12:1–12. 35948584 10.1038/s41598-022-17917-2 PMC9365789

[124] Wang M, Li L, Qian J, Wang N, Bao J, Lu J, et al. Periodontitis salivary microbiota exacerbates nonalcoholic fatty liver disease in high-fat diet-induced obese mice. iScience. 2023;26:106346 10.1016/J.ISCI.2023.106346. 36968080 10.1016/j.isci.2023.106346 PMC10031158

[125] Joo K, Kang YW, Moon SY, Baek YH, Son M. Association between nonalcoholic fatty liver disease scores and chronic periodontitis: a retrospective cohort study. J Periodontol. 2024. 10.1002/JPER.24-0171. 10.1002/JPER.24-0171 38971999

[126] Kumar PS. Microbial dysbiosis: the root cause of periodontal disease. J Periodontol. 2021;92:1079–87. 34152022 10.1002/JPER.21-0245

[127] Sedghi LM, Bacino M, Kapila YL. Periodontal disease: the good, the bad, and the unknown. Front Cell Infect Microbiol. 2021;11:766944. 34950607 10.3389/fcimb.2021.766944 PMC8688827

[128] Martinon P, Fraticelli L, Giboreau A, Dussart C, Bourgeois D, Carrouel F. Nutrition as a key modifiable factor for periodontitis and main chronic diseases. J Clin Med. 2021;10:197. 33430519 10.3390/jcm10020197 PMC7827391

[129] Woelber JP, Gärtner M, Breuninger L, Anderson A, König D, Hellwig E, et al. The influence of an anti-inflammatory diet on gingivitis. A randomized controlled trial. J Clin Periodontol. 2019;46:481–90. 30941800 10.1111/jcpe.13094

[130] Woelber JP, Bremer K, Vach K, König D, Hellwig E, Ratka-Krüger P, et al. An oral health optimized diet can reduce gingival and periodontal inflammation in humans - a randomized controlled pilot study. BMC Oral Health. 2016;17:1–8. 27460471 10.1186/s12903-016-0257-1 PMC4962497

[131] Tennert C, Reinmuth AC, Bremer K, Al-Ahmad A, Karygianni L, Hellwig E, et al. An oral health optimized diet reduces the load of potential cariogenic and periodontal bacterial species in the supragingival oral plaque: a randomized controlled pilot study. Microbiologyopen. 2020;9:e1056. 32419378 10.1002/mbo3.1056 PMC7424251

[132] Woelber JP, Bartha V, Baumgartner S, Tennert C, Schlagenhauf U, Ratka-Krüger P, et al. Is diet a determining factor in the induction of gingival inflammation by dental plaque? a secondary analysis of clinical studies. Nutrients. 2024; 16. 10.3390/NU16070923. 10.3390/nu16070923 PMC11013428 38612955

[133] Dang TS, Walker M, Ford D, Valentine RA. Nutrigenomics: the role of nutrients in gene expression. Periodontol. 2014;64:154–60. 10.1111/prd.12001 24320962

